# Hodge theory-based biomolecular data analysis

**DOI:** 10.1038/s41598-022-12877-z

**Published:** 2022-06-11

**Authors:** Ronald Koh Joon Wei, Junjie Wee, Valerie Evangelin Laurent, Kelin Xia

**Affiliations:** Division of Mathematical Sciences, School of Physical and Mathematical Sciences, Nanyang Technological University, Singapore, 637371 Singapore

**Keywords:** Computational biology and bioinformatics, Mathematics and computing

## Abstract

Hodge theory reveals the deep intrinsic relations of differential forms and provides a bridge between differential geometry, algebraic topology, and functional analysis. Here we use Hodge Laplacian and Hodge decomposition models to analyze biomolecular structures. Different from traditional graph-based methods, biomolecular structures are represented as simplicial complexes, which can be viewed as a generalization of graph models to their higher-dimensional counterparts. Hodge Laplacian matrices at different dimensions can be generated from the simplicial complex. The spectral information of these matrices can be used to study intrinsic topological information of biomolecular structures. Essentially, the number (or multiplicity) of *k*-th dimensional zero eigenvalues is equivalent to the *k*-th Betti number, i.e., the number of *k*-th dimensional homology groups. The associated eigenvectors indicate the homological generators, i.e., circles or holes within the molecular-based simplicial complex. Furthermore, Hodge decomposition-based HodgeRank model is used to characterize the folding or compactness of the molecular structures, in particular, the topological associated domain (TAD) in high-throughput chromosome conformation capture (Hi-C) data. Mathematically, molecular structures are represented in simplicial complexes with certain edge flows. The HodgeRank-based average/total inconsistency (AI/TI) is used for the quantitative measurements of the folding or compactness of TADs. This is the first quantitative measurement for TAD regions, as far as we know.

## Introduction

With the help from various experimental tools, including mass spectrometry, X-ray, Nuclear magnetic resonance (NMR), and Cryogenic electron microscopy (cryo-EM), there is an accumulation of biomolecular structure data in various databanks, such as Protein Data Bank (PDB) and Electron Microscopy Data Bank (EMDB). The availability of these large amount of biomolecular data provides great opportunities for researchers in data sciences^1^. Due to the biomolecular structure–function relationships, a better description and characterization of biomolecular structures can help to improve the accuracy of models for biomolecular functions^2^. For quantitative structure-activity/property relationship (QSAR/QSPR) and machine learning models, structure-based molecular descriptors are of essential importance^3,4^. Structural features that characterize deep, intrinsic and fundamental molecular properties have better learning accuracy, as they have a better transferability^5,6^. Recently, Hodge theory-based persistent spectral models, including persistent spectral graph^7,8^, persistent spectral simplicial complex^9^, and persistent spectral hypergraph^10^, have been used in protein B-factor and protein-ligand binding affinity prediction. Different from traditional graph-based molecular descriptors, Hodge theory-based molecular features incorporate both topological and geometric information and provide a balance between structure complexity and data simplification^9^.

Mathematically, as a bridge between differential geometry, algebraic topology, and functional analysis, Hodge theory unveils the fundamental relations of differential forms^11,12^. Based on de-Rhams cohomology and Hodge star operator, Hodge Laplacian (HL) operator is defined from differential forms on Riemannian manifolds^13^. The kernel of the HL operator induces harmonic forms, which reflect the homology of the manifold. Furthermore, Hodge theory provides an orthogonal decomposition of the differential forms, known as Hodge decomposition^14^. Hodge theory, which was originally defined on the Riemannian manifolds, can be viewed as “differentiable Hodge theory”. A “continuous Hodge theory” is proposed by the generalization of Hodge theory onto metric spaces^15^.

Computationally, combinatorial Hodge theory or discrete Hodge theory has been proposed^16–23^. Essentially, this discrete version can be viewed as part of exterior calculus and discrete differential geometry. To avoid confusion, there are two components of discrete Hodge theory, i.e., Hodge Laplacian matrices and discrete Hodge decomposition^18,24,25^. HL matrix (or combinatorial Laplacian matrix) is constructed on simplicial complex^16–18^ and hypergraph^26–30^. It can be regarded as a generalization of the graph Laplacian matrix into its higher-dimensional counterpart. The spectral information of HL matrices contains the topological information of the underlying structures. In particular, the multiplicity of the zero eigenvalue of HL matrices corresponds to Betti numbers, i.e., number of cycles or loops. Eigenvectors from zero eigenvalues are related to the homology generators. Geometrically, the components with large-absolute-values of zero-eigenvalue-related eigenvectors concentrate around cycles or loops of structures^31^. Furthermore, discrete Hodge decomposition models have been used in statistical ranking^25^ and game theory^32^. HodgeRank models have been developed for ranking incomplete or imbalanced data from e-commerce and internet applications^25^. The essential idea is to reveal ranking information from edge flows, which represent difference between pairs of vertices and thus are pairwise ranking. In particular, an edge flow can be decomposed into three orthogonal components, a gradient flow that represents the optimal global ranking, a curl flow (locally cyclic), and a harmonic flow (locally acyclic but globally cyclic). The curl flow and harmonic flow are divergence-free flow (cyclic) that measures the ranking inconsistency. Recently, a five-component orthogonal decomposition model has been proposed^33,34^. It can split a discrete vector field, which is represented as discrete differential forms, into two potential fields, as well as three additional harmonic components. The model has been successfully used for the analysis of biological macromolecules and subcellular organelles, in particular, the flexibility and normal modes of molecular structures^33,34^.

Biomolecular folding and compactness are of great importance to their intrinsic functions and properties. The importance of protein folding cannot be overstated. Ill-folded proteins can lead to various diseases, such as Alzheimer’s disease, mad cow disease, and Parkinson’s disease. Further, as the most important genetic information, DNA also forms highly complicated structures. In eukaryote cells, DNA molecules bind with histone proteins to form nucleosomes. A nucleosome has a core region and a linker region. The core region consists of around 146 DNA base pairs wrapped around eight histone proteins in a left-handed superhelical pattern. The core regions are connected to nucleosome linker DNA, which can be as long as 80 DNA base pairs. Geometrically, the core region looks like a “bead” and the linker DNA like a “string” between “beads”. The nucleosome “beads-on-a-string” chains fold into chromatin fibres, which are at the size of 30-nanometer. Moreover, these chromatin fibres will further fold into highly complicated and compacted chromosome structures. Folding properties and compactness are key to the understanding of chromosomal structures and their functions. As one of the most complex and important cellular entities, chromosomes are the physical realization of genetic information^35–41^, and play important roles in various biological functions^42,42–45^, such as DNA replication, DNA transcription, repair of DNA damage, chromosome translocation, the development of epigenetic organizations, the regulation of genome functions, and the epigenetic inheritance of various cell states. Various experimental tools are developed to understand the chromosome folding and compactness, among them is the chromosome conformation capture (3C) technique^46,47^ and its derived methods, including chromosome conformation capture-on-chip (4C)^48,49^, chromosome conformation capture carbon copy (5C)^50^ and high-throughput chromosome conformation capture (Hi-C)^51^. These experimental techniques have been developed and begun to uncover general features of genome organization^51–59^. In particular, the modeling and analysis Hi-C data have indicated a special folding pattern known as topologically associating domains (TADs)^52,53^. TADs are highly-compacted and folded chromosome regions with a size from about 200 kilobases (Kb) to 2 megabases (Mb). Computationally, they are defined to be the contiguous square regions along the diagonal Hi-C maps with large contact values. These square regions are found to be very consistent between different cell types and species and their spatial distributions are highly correlated with many genomic features such as histone modifications, coordinated gene expression, lamina, and DNA replication timing. Various algorithms and software are designed to identify these TAD regions from Hi-C data, such as hidden Markov model (HMM)^52^, Armatus^60^, HiCseg^61^, spectral models TADs^62,63^. All these models focus on matrix or graph segmentation and optimization of the block or square regions. No rigorous mathematical definitions or models are proposed to uniquely define TAD regions.

In this paper, we analyze biomolecular data and Hi-C data with Hodge theory-based models. The Hodge Laplacian-based spectral information is used for biomolecular structure analysis. The multiplicity of the zero eigenvalue represents Betti numbers^18^. Eigenvectors are used to identify homology and non-homology generators. Geometrically, homology generators (eigenvectors from zero eigenvalues) correspond to the cycle structures within the data. Non-homology generators can be used in clustering (spectral clustering) and community detection^17,21^. Furthermore, eigendecomposition-based HodgeRank model can be used in biomolecular structure folding analysis. Different from general molecules from materials and chemistry, biomolecules are three-dimensional structures that are folded from one or several individual chains. In our model, molecular structures are represented in simplicial complexes with certain edge flows. The average/total inconsistency (AI/TI) is used as a quantitative measurement of the folding or compactness of structures. More specifically, we incorporate coordinate-related structural information into edge flow terms. The curl flow terms and harmonic flow terms, from the Hodgerank decomposition, characterize the local and non-local compactness/folding properties. For Hi-C data, an important issue is the topological associated domain (TAD). Even though various elegant algorithms and methods have been proposed for the identification of TADs, there is no quantitative way to characterize how likely a certain region in Hi-C data is a TAD. Here we generate simplicial complexes from Hi-C contact matrix, and use AI as a way to quantitatively measure TAD likelihood. The AI characterizes the compactness or folding of the structure. We have validated the model with experimental Hi-C data from human embryonic stem cells chromosome 10. The predictions from our models are highly consistent with the TAD patterns.

## Methods

We use discrete Hodge models, including Hodge Laplacian and Hodge decomposition, for biomolecular structure representation and characterization. Different from previous graph-based models, molecular structures are represented as simplicial complexes. Algebraic tools from chain groups, homology groups, boundary operators, Laplacian matrices, and orthogonal decomposition, are used to reveal deeper geometric and topological properties of these molecular structures.

### Topological representations for biomolecules

**Figure 1:**
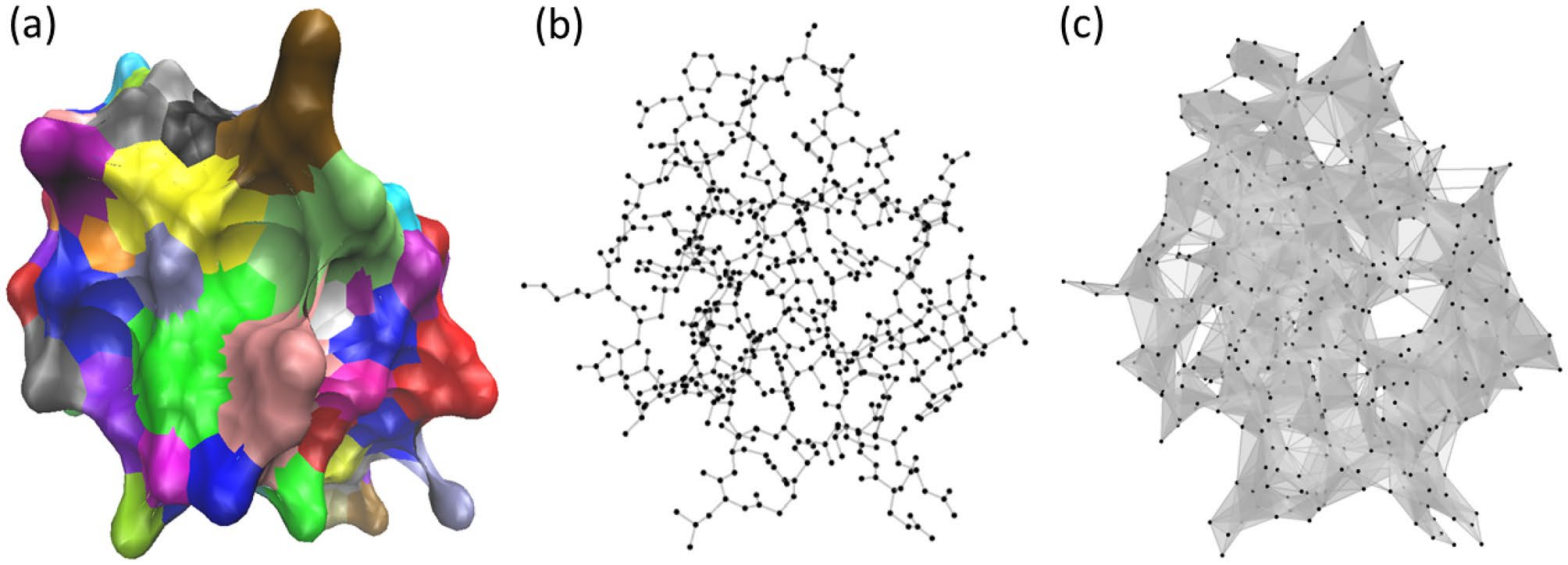
The comparison of molecular graph and simplicial complex representations for a protein (ID:2OFS). (**a**) Surface representation for protein 2OFS. (**b**) A graph representation for protein 2OFS. (**c**) The simplicial complex model for protein 2OFS. A simplicial complex can be viewed as a generalization of graph into its higher dimensional counterpart. Vertices (0-simplexes) and edges (1-simplexes) from graphs can be extended to higher dimensional elements, including triangles (2-simplexes) and tetrahedrons (3-simplexes).

The biomolecular topology is of essential importance for biomolecular flexility, dynamics and functions. For instance, molecular dynamic (MD) force field involves geometric/topological features, such as bond length, bond angle, dihedral angle, and other graph-based properties^64^. In fact, graphs or networks are the most frequently used models for the representation of molecular structures from materials, chemistry and biology^3,4^. Mathematically, a *graph* $G=(V,E)$ contains the *vertex set* $V=\{ v_i: 1 \le i \le N\}$ and the *edge set* $E=\{(v_i,v_j): 1 \le i,j \le N \}$. Generally speaking, graph representations only characterize the zero and one-dimensional information within the structure. A simplicial complex is a generalization of graphs into their higher dimensional counterpart. The most commonly used simplicial complexes are triangle meshes and tetrahedron meshes. A general simplicial complex is composed of simplexes. Based on simplicial complexes, various algebraic groups, boundary operators, and Hodge Laplacian matrices can be defined.

Let *d*, *k* be any two positive integers and $U=\{u_0,u_1,\ldots ,u_k \}$ be a collection of points in ${\mathbb {R}}^d.$ We say that this collection of points are *affinely independent* if the set $\{u_i-u_0 \}_{i=1}^{k}$ is linearly independent. A point $x \in {\mathbb {R}}^{d}$ is said to be an *affine combination* of points in *U* if it can be written as a linear combination of points in *U* whose coefficients sum to 1, that is,

$$x = \sum_{i=0}^{k} \lambda_{i} u_{i},$$

for some $\lambda _i \in {\mathbb {R}}$ and $\sum_{i=0}^k \lambda _i=1.$ If $\lambda _i \ge 0$ also holds, then *x* is said to be a *convex combination* of points in *U*. The *convex hull* of *U* is the set of all convex combinations of points in *U*. The fundamental building blocks of simplical complex are simplexes.

Let $U=\{u_0,u_1,\ldots ,u_k \}$ be an affinely independent set of $k+1$ points in ${\mathbb {R}}^d$. A *k-simplex* $\sigma ^k$ is the convex hull of *U*, denoted by $[u_0,u_1,\ldots ,u_k]$. The *dimension* of $\sigma ^k$ is *k*. Geometrically, a 0-simplex is simply a point, an 1-simplex is called an edge, a 2-simplex is called a triangle and a 3-simplex is called a tetrahedron. A *face* $\tau$ of a *k*-simplex $\sigma ^k$ is the convex hull of a non-empty subset *A* of *U*, denoted by $\tau \subset \sigma ^k$. An oriented *k*-simplex is a *k*-simplex with an orientation, i.e., a sequence arrangement of its vertices. If two *k*-simplexes $\sigma _1^k$ and $\sigma _2^k$ are of the same orientation, they are denoted as $\sigma _1^k \sim \sigma _2^k$. Two simplexes $\sigma _1^k$ and $\sigma _2^k$ are *upper adjacent* and denoted as $\sigma _1^k \frown \sigma _2^k$, if they are faces of a common $(k+1)$-simplex, and they are *lower adjacent* and denoted as $\sigma _1^k \smile \sigma _2^k$, if they share a common $(k-1)$-simplex as their face. For these two oriented *k*-simplices, if the orientations of their common lower simplex are the same, they are called a *similar common lower simplex* and denoted by $\sigma _1^k \smile \sigma _2^k$ and $\sigma _1^k \sim \sigma _1^k$. Otherwise, it is called a *dissimilar common lower simplex* and denoted by $\sigma _1^k \smile \sigma _2^k$ and $\sigma _1^k \not \sim \sigma _2^k$. The *(upper) degree* of a *k*-simplex $\sigma ^k$, denoted by $d(\sigma ^k)$, is the number of $(k+1)$-simplices of which $\sigma ^k$ is a face.

A *simplicial complex* *K* is a finite collection of simplices that satisfy two conditions. Firstly, any face of a simplex in *K* is also in *K*. Secondly, the intersection of any two simplices in *K* is either empty or a face of both. A *simplicial* *k-complex* is a simplicial complex where the largest dimension of simplices in *K* is *k*. Figure 1 illustrates the comparison between a graph and a simplicial complex for a protein (ID:2OFS). For the graph model, vertices represent molecular atoms and the edges are for covalent-bonds. The simplicial complex is constructed using Vietoris-Rips complex. Essentially, a cutoff-distance of 4.0Å is used and a *k*-simplex is formed among $k+1$ vertices whose pair-wise distances are all smaller than 4.0Å.

## Hodge Laplacian and Hodge decomposition

Hodge Laplacian matrices of different dimensions can be constructed on a simplicial complex. A *k*-th dimensional HL matrix characterizes topological connections between *k*-th simplexes. Note that the graph Laplacian, which is 0-th dimensional HL, characterizes relations between vertexes (0-simplexes).

### Hodge Laplacian model

#### Mathematical background for Hodge Laplacian

The *k*th chain group $C_k(K)$ of a simplicial complex *K* over some field ${\mathbb {F}}$ is a vector space over the ${\mathbb {F}}$ whose basis is the set of *k*-simplices of the simplicial complex *K*. Elements of $C_k(K)$ are called *k*-chains. The *dual* of $C_k(K),$ denoted by $C^k(K),$ is the set of all linear functionals on $C_k(K)$:

$$C^k(K)=\big \{\phi : C_k(K) \rightarrow {\mathbb {F}}\, : \, \phi \, {\text{is linear}} \big \}.$$

$C^k(K)$ is called the *k-th cochain group* and its elements are called *k-cochains*. Boundary operators are defined on both the chain and cochain groups. The *boundary map* $\partial _k:C_k(K) \rightarrow C_{k-1}(K)$ is a linear transformation which acts on a *k*-simplex $\sigma ^k=[u_0,u_1,\ldots ,u_k]$ as follows

$$ {}\partial _k([u_0,u_1,\ldots ,u_k])=\sum _{i=0}^k(-1)^i[u_0,\ldots ,u_{i-1},u_{i+1},\ldots ,u_k]. $$

The *coboundary map* $\delta _k:C^k(K) \rightarrow C^{k+1}(K)$ is a linear transformation defined as follows: for a linear functional $\phi \in C^k(K)$ and a $k+1$-simplex $\sigma ^{k+1}=[u_0,u_1,\ldots ,u_{k+1}]$,

$$ \delta _k(\phi )(\sigma ^{k+1})=\sum _{i=0}^{k+1}(-1)^i\phi ([u_0,\ldots ,u_{i-1},u_{i+1},\ldots ,u_{k+1}]). $$

The boundary map gives rise to a *chain complex*, which is a sequence of chain groups connected by boundary maps as follows:

$$\begin{aligned} 0 \rightarrow C_{n}(K)\rightarrow \cdots \xrightarrow {\partial _{k+1}}C_k(K)\xrightarrow {\partial _k} C_{k-1}(K)\cdots \xrightarrow {\partial _2}C_1(K)\xrightarrow {\partial _1}C_0(K)\rightarrow 0. \end{aligned}$$

Similar to the boundary map giving rise to the chain complex, the coboundary operator gives rise to a *cochain complex*:

$$\begin{aligned} 0 \leftarrow C^{n}(K)\leftarrow \cdots \xleftarrow {\delta _{k}}C^k(K)\xleftarrow {\delta _{k-1}} C^{k-1}(K)\cdots \xleftarrow {\delta _1}C^1(K)\xleftarrow {\delta _0}C^0(K)\leftarrow 0. \end{aligned}$$

Since $C_k(K)$ and $C^k(K)$ are finite-dimensional, there exists unique matrix representations for $\partial _k$ and $\delta _k$. We have some useful relations regarding matrix representations of $\partial _k$ and $\delta _k$ ($A^T$ represents the transpose of a matrix *A*):

- For all $$k \ge 0$$, $$\partial _{k+1}^T=\delta _k$$,
- $$\partial _k^T=\partial _k^*$$,
- $$\delta _k^T=\delta _k^*.$$

Here, $\delta _k^*:C^{k+1}(K)\rightarrow C^k(K)$ is the *adjoint/transpose map* of $\delta _k$ where

$$ \langle \delta _k(f), g \rangle =\langle f, \delta _k^*(g) \rangle , $$

for every $f \in C^k(K)$, $g \in C^{k+1}(K)$ and a suitable inner product $\langle \, , \rangle$ for $C^k(K)$ and $C^{k+1}(K).$ The adjoint of the boundary operator $\partial _k$, $\partial _k^*$ is also defined analogously. These relations above allow us to work unilaterally from the boundary operator’s perspective, which is the easiest to compute amongst the two.

The *k*-dimensional *combinatorial Laplacian* is the linear operator $\Delta _k:C^k(K) \rightarrow C^k(K)$ is defined as follows:

$$\begin{aligned} \Delta _k= {\left\{ \begin{array}{ll} \delta _k^*\circ \delta _k+\delta _{k-1}\circ \delta _{k-1}^* &{} \text {if } k \ge 1, \\ \delta _k^*\circ \delta _k &{} \text {if } k=0. \end{array}\right. } \end{aligned}$$

The case where $k=0$ gives rise to the expression of the well-known graph Laplacian.

### Discrete Hodge Laplacian

The boundary operator $\partial _k$ has a unique matrix representation. Given a simplicial complex *K*, the *k-th boundary matrix* $\pmb {B}_k$ is defined as,

$$ (\pmb {B}_k)_{ij}= {\left\{ \begin{array}{ll} 1 &{}\text {if } \sigma _i^{k-1} \subset \sigma _j^{k} ~\text {and }~ \sigma _i^{k-1} \sim \sigma _j^{k}, \\ -1 &{}\text {if } \sigma _i^{k-1} \subset \sigma _j^{k} ~\text {and }~ \sigma _i^{k-1} \not \sim \sigma _j^{k}, \\ 0 &{}\text {if } \sigma _i^{k-1} \not \subset \sigma _j^{k}. \end{array}\right. } $$

Here $\sigma _i^{k-1}$ is the *i*-th $(k-1)$-simplex and $\sigma _j^{k}$ is the *j*-th *k*-simplex.

Given that the highest order of the simplicial complex *K* is *n*, the *k*th Hodge Laplacian (or combinatorial Laplacian) matrix $\pmb {L}_k$ of *K* is

$$ \pmb {L}_k= {\left\{ \begin{array}{ll} \pmb {B}_n^T\pmb {B}_n &{} \text {if } k=n, \\ \pmb {B}_k^T\pmb {B}_k+\pmb {B}_{k+1}\pmb {B}_{k+1}^T &{} \text {if } 1 \le k < n, \\ \pmb {B}_1\pmb {B}_1^T &{} \text {if } k=0. \end{array}\right. } $$

These *k*-th HL matrices can also be expressed in terms of simplex relations. When $k=0$,

$$ (\pmb {L}_0)_{ij}= {\left\{ \begin{array}{ll} d(\sigma _i^{0}) & {}{\text {if }}\ i=j, \\ -1 & {} \text {if } i \ne j\ {\text {and }} \sigma _i^{0} \smallfrown \sigma _j^{0}, \\ 0 & {} {\text {if }} i \ne j\ {\text {and }} \sigma _i^{0} / \kern-18pt \smallfrown \sigma _j^{0}. \end{array}\right. }$$

The HL matrix $\pmb {L}_0$ is the graph Laplacian matrix. When $k>0$,

$${({\pmb{L}_k})_{ij}}= {\left\{ \begin{array}{ll} d(\sigma_{i}^{k})+ k+1 &\text {if } i=j, \\ 1 & \text {if } i \ne j, \; {\sigma_{i}^{k}}\; /\kern-16pt \smallfrown {\sigma_{j}^{k}}, {\sigma_{i}^{k}} \smile {\sigma_{j}^{k}}~\text {and } {\sigma _i^k} \sim {\sigma_{j}^k}, \\ -1&\text {if } i \ne j, \; \sigma_{i}^{k}\; /\kern-16pt \smallfrown \sigma_{j}^{k}, \sigma_{i}^{k} \smile \sigma_{j}^{k} \; \text {and } \sigma_{i}^k /\kern-16pt \sim \sigma_{j}^k, \\ 0 &\text {if } i \ne j, \; \sigma_{i}^{k} \smallfrown \sigma_{j}^{k} \; \text {or }\; \sigma_{i}^{k} /\kern-16pt \smile \sigma_{j}^{k}. \end{array}\right. }$$

Mathematically, the eigenvalues of HL matrices are independent of the choice of the orientation^18^.

### Hodge decomposition model

Hodge decomposition is an orthogonal decomposition of a vector field into gradient part, harmonic part and curl part. Hodge decomposition has been used in fluid mechanics, data analysis, game theory and molecular dynamics^25,32–34^.

Mathematically, from the 1st cochain group, if we denote,

$$ {\text {ker}}(\Delta _1)={\text {ker}}(\delta _1)\cap {\text {ker}}(\delta _0^*), $$

the Hodge decomposition^25^ can be expressed as follows,

$$ C^1(K)={\text {Im}}(\delta _0)\oplus {\text {ker}}(\Delta _1)\oplus {\text {Im}}(\delta _1^*). $$

Geometrically, the cochain $C^1(K)$ can be viewed as *edge flows* as it consists of all scalar functions on the 1-simplices (edges). The term ${\text {ker}}(\delta _1)$ can be regarded as *gradient flows*, term ${\text {ker}}(\Delta _1)$ can be regarded as *harmonic flows*, and term ${\text {Im}}(\delta _1^*)$ can be regarded as *curl flows*.

### Discrete Hodge decomposition and HodgeRank

Computationally, Hodge decomposition-based surface vector field analysis^14^ have received a lot of attention, and found various applications in geometric processing, computer graphs, and fluid dynamics analysis. Different from these 2D surface or 3D domain-based vector decomposition models, a simplicial complex-based Hodge decomposition model, known as HodgeRank, has been proposed for statistical ranking^25^.

In HodgeRank^25^, an edge flow value *Y* on an edge is regarded as a ranking order, that is if the flow goes from vertex $i_1$ to vertex $i_2$, then the score is higher at $i_1$ than $i_2$ (as flow goes from higher “place” to lower “place”). The edge flow from vertex $i_1$ to vertex $i_2$ is denoted as $Y_{[i_1, i_2]}$. If the rank value for $i_1$ is a scale with value $f_{i_1}$ and for $i_2$ is $f_{i_2}$, then $Y_{[i_1, i_2]}=f_{i_1}-f_{i_2}$. In this way, gradient flows $Y^g$ are globally consistent in terms of ranking, as they always go from higher values to low values^25^. In contrast, harmonic flows $Y^h$ and curl flows $Y^c$ are inconsistent in ranking models^25^. In both terms, the flows can travel from one vertex to some other vertices and then return to the same exact vertex. This is problematic for ranking, as it means a “large” value can keep on decreasing to “small” values, but still return to the same value. The harmonic flows are globally inconsistent and curl flows are locally inconsistent.

Given the edge flow values *Y*, HodgeRank gives the gradient flow term $Y^g$, the curl flow term $Y^c$, and the harmonic flow term $Y^h$. The detailed algorithm for Hodge decomposition is listed in Algorithm 1.
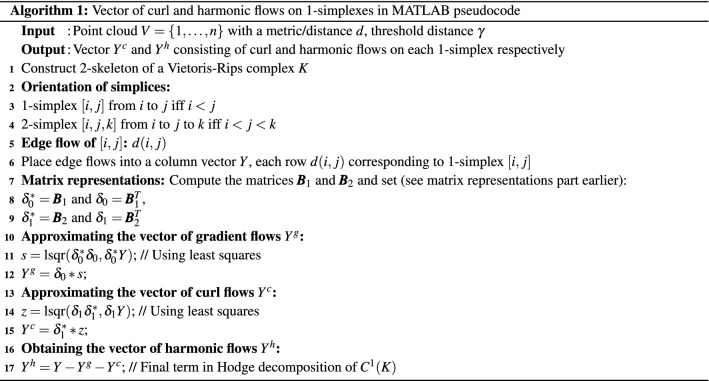


Further, *total inconsistency (TI)* can be defined as follows,

1 $$\begin{aligned} \mathrm{TI}=\sum _{[i,j]\in K}\left| \frac{(Y^c+Y^h)_{[i,j]}}{Y_{[i,j]}}\right| , \end{aligned}$$

here [*i*, *j*] is oriented 1-simplex in the simplicial complex *K*, the term $Y_{[i,j]}$ is the orginal vector flow on 1-simplex [*i*, *j*], and the term $(Y^c+Y^h)_{[i,j]}$ represents the sum of the curl and harmonic flows on the 1-simplex [*i*, *j*].

To compare the structures with different sizes, one can use *average inconsistency (AI)*,

2 $$\begin{aligned} \mathrm{AI}=\frac{1}{N}\sum _{[i,j]\in K}\left| \frac{(Y^c+Y^h)_{[i,j]}}{Y_{[i,j]}}\right| , \end{aligned}$$

here *N* is the total number of 0-simplexes (vertices) in the simplicial complex. We also note that the TI/AI indices does not depend on the ordering of vertices, as the number of edges and triangles in a Vietoris-Rips simplicial complex with a fixed threshold distance $\gamma$ is invariant under the renumbering of data points, and the set of values in the vectors of curl and harmonic flows are each uniquely determined by $\gamma$.

### Hodge-theory-based biomolecular structure analysis

We use Hodge Laplacian and Hodge decomposition models to analyze biomolecular structures. Both homological and non-homological eigenvectors from Hodge Laplacian can be used in the different types of spectral clustering. The Hodge decomposition-based Hodgerank model can be used in the systematic characterization of biomolecular folding and compactness, in particular, the analysis of TAD regions from Hi-C data.

**Figure 2:**
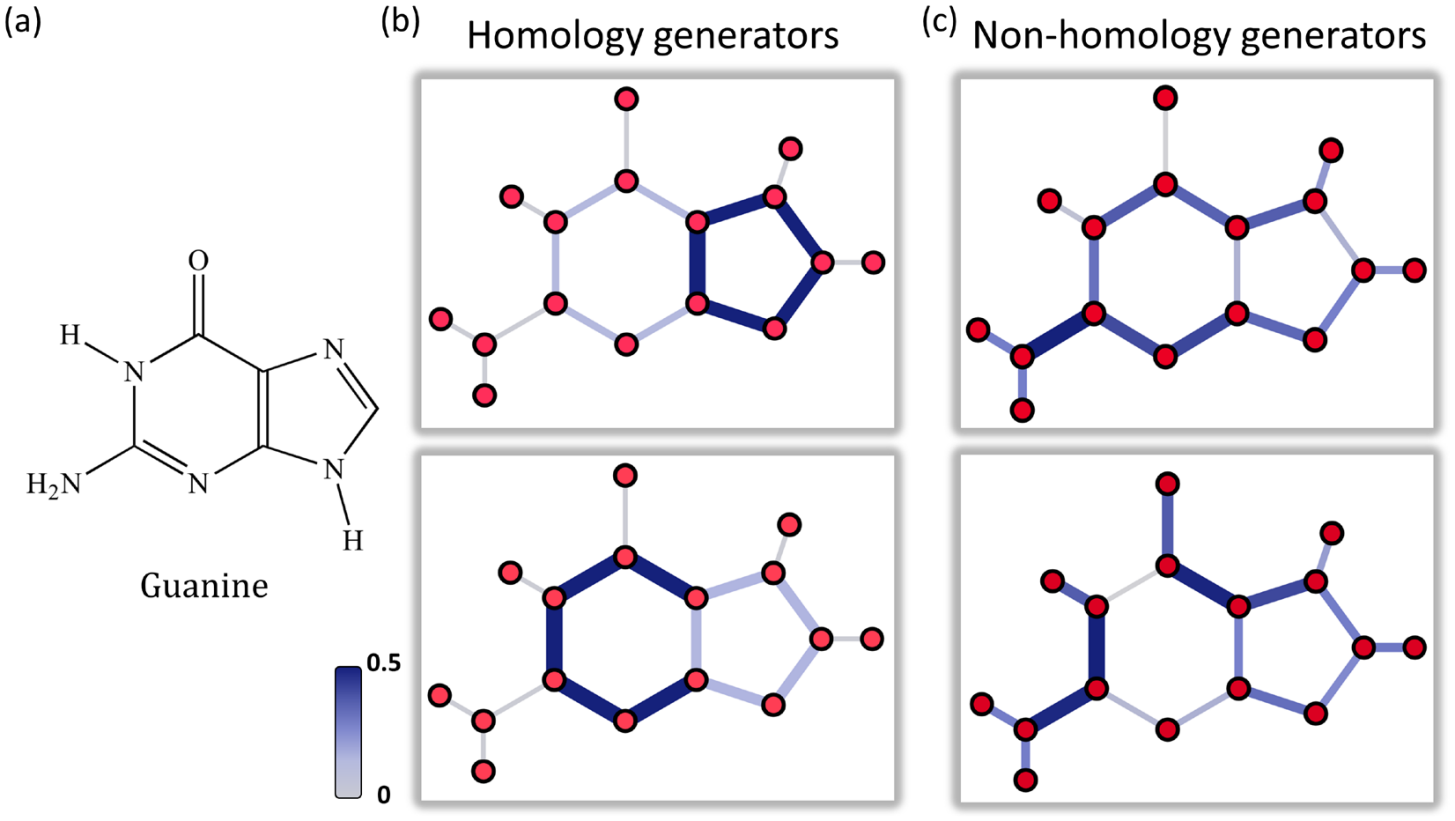
The illustration of HL-based structure analysis. (**a**) The graph representation for Guanine. (**b**) The zero-eigenvalue-related eigenvectors are homology generators. Geometrically, their largest absolute values indicate the associated loop structures. (**c**) Nonzero-eigenvalue-related eigenvectors are more related to domain, cluster and community structures.

#### Hodge Laplacian-based biomolecular structure analysis

The multiplicity of the zero eigenvalue, i.e., the total number of zero eigenvalues, of $\mathbf{L}_k$ is the *k*-th Betti number $\beta _k$. Geometrically, $\beta _0$ is the number of connected components, $\beta _1$ is the number of circles or loops, and $\beta _2$ is the number of cavities. Moreover, the zero eigenvalue related eigenvectors are related to homology generators. They can be used to identify the associated topological features, such as circles, loops, and voids in the structures. The eigenvectors from nonzero eigenvalues are related to clusters and communities within the data, and can be used for spectral clustering.

Figure 2 illustrates $\mathbf{L}_1$-based eigenvectors for Guanine structures. The absolute values of $\mathbf{L}_1$ eigenvectors are plotted on the edges and represented by colors.Two homology generators, i.e., eigenvectors from the zero-eigenvalue, are considered. It can be seen that for each homology generator, their largest absolute values are all concentrated around a loop structure, which is either a pentagon ring or a hexagon ring. In contrast, for the two (non-homological) eigenvectors that are from the non-zero-eigenvalues, their values can be used to identify domains or clusters.

#### HodgeRank-based biomolecular structure analysis

Recently, Hodge decomposition for vector fields over 3D bounded domains has been systematically explored and been applied to biomolecular dynamic analysis^33,34^.

The essential idea is to use HodgeRank-based TI/AI indices as a way to measure the folding, curvedness and compactness of the biomolecular structures. In our model, edge flows represent distance relations between biomolecular atoms. Note that biomolecular atoms have a unique ordering or sequence. For instance, DNAs are double helix structures from the gene sequence, and proteins are from peptide sequences. The gene or peptide sequence provides a natural ordering of the atoms in a biomolecule. In this way, even though the biomolecules have highly complicated 3D structures, their atoms, in particular the backbone atoms, can be systematically arranged into a unique sequence (following their gene sequence). Furthermore, the inconsistence from edge flows can be used to model Euclidian distances deviated from the straight lines. For two vertices $i_1$ and $i_2$ with coordinate $\mathbf{r}_{i_1}$ and $\mathbf{r}_{i_2}$, the edge flow $Y_{[i_1, i_2]}$ is defined as,

3 $$\begin{aligned} Y_{[i_1, i_2]}= {\left\{ \begin{array}{ll} |\mathbf{r}_{i_1}-\mathbf{r}_{i_2}| &{} i_1<i_2, \\ -|\mathbf{r}_{i_1}-\mathbf{r}_{i_2}| &{} i_1>i_2. \end{array}\right. } \end{aligned}$$

Note that edge flows are always positive if they follow the chain sequence. More specifically, if vertex $i_2$ comes later than $i_1$ along the chain sequence, then $Y_{[i_1, i_2]}$ is always positive, otherwise the edge flow is negative.

**Figure 3:**
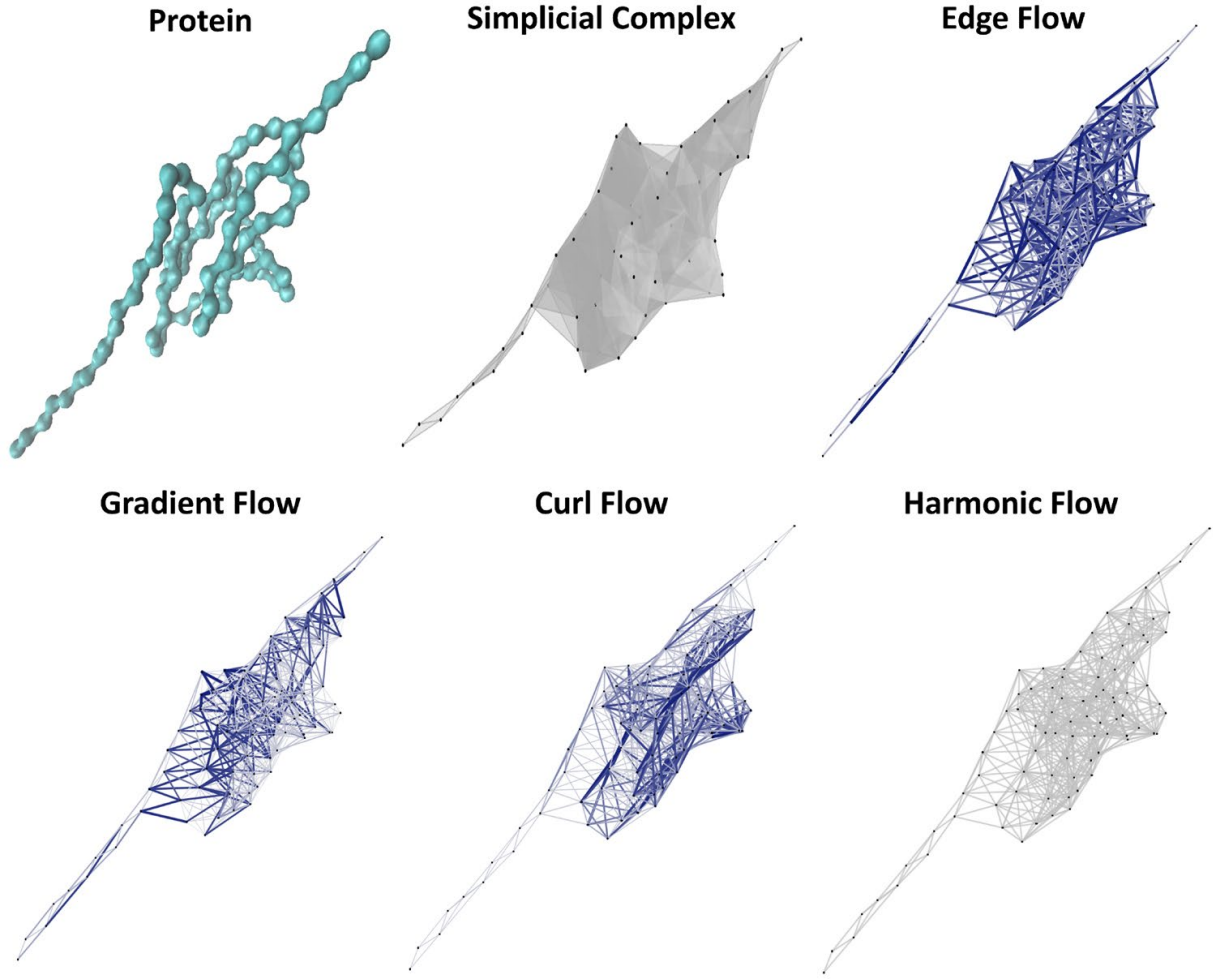
The illustration of the different flow terms, i.e., gradient, curl, and harmonic, for a partially-folded protein. Only $C_\alpha$ atoms are considered and the protein configuration is taken from the SMD simulation^65^. The simplicial complex is generated with a cutoff at 11 Å. It can be seen that most of curl terms with larger values are concentrated near highly-packed regions. All harmonic terms are zero, since there is no harmonic flows (no 1D harmonic circles in the simplicial complex).

Motivated by the triangle inequality definition, we propose to use local inconsistence to measure the curvedness of the biomolecular chains. More specifically, if three vertices $i_1$, $i_2$ and $i_3$ are located in a straight line, we should always have the sum $Y_{[i_1, i_2]}+Y_{[i_2, i_3]}+Y_{[i_3, i_1]}=0$, meaning there is no curvedness or folding. In contrast, if the sum is nonzero, there will be a deviation from the straight line. More generally, if the whole chain is a straight line, the edge flows defined above will only have gradient terms. Both harmonic flows and curl flows will be zero. In contrast, if a chain is folded, the harmonic flows and curl flows are nonzero and can be used to characterize the curvedness, folding and compactness of structures. In Fig. 3, we illustrate different flow terms of the simplicial complexes generated from an partially-folded protein structure (details in “Protein folding analysis”). It can be seen that the large-valued curl flow terms are all concentrated in the highly-packed or folded regions. The harmonic flow terms are all zero as there is no 1D harmonic circles in the simplicial complexes. It is worth mentioning that the curl flow terms are only defined on 2-simplexes (triangles), thus there will be no curl flow terms if there is no 2-simplexes.

## Results

In this section, we apply Hodge Laplacian and HodgeRank models into biomolecular data analysis and Hi-C data analysis. HL-based eigenvectors are used to reveal cycle or loop structures within molecules. Furthermore, Hodge decomposition-based TI/AI indices are used for protein, DNA and chromatin folding analysis.

### Hodge-theory-based biomolecular data analysis

#### HL-based biomolecular structure analysis

**Figure 4:**
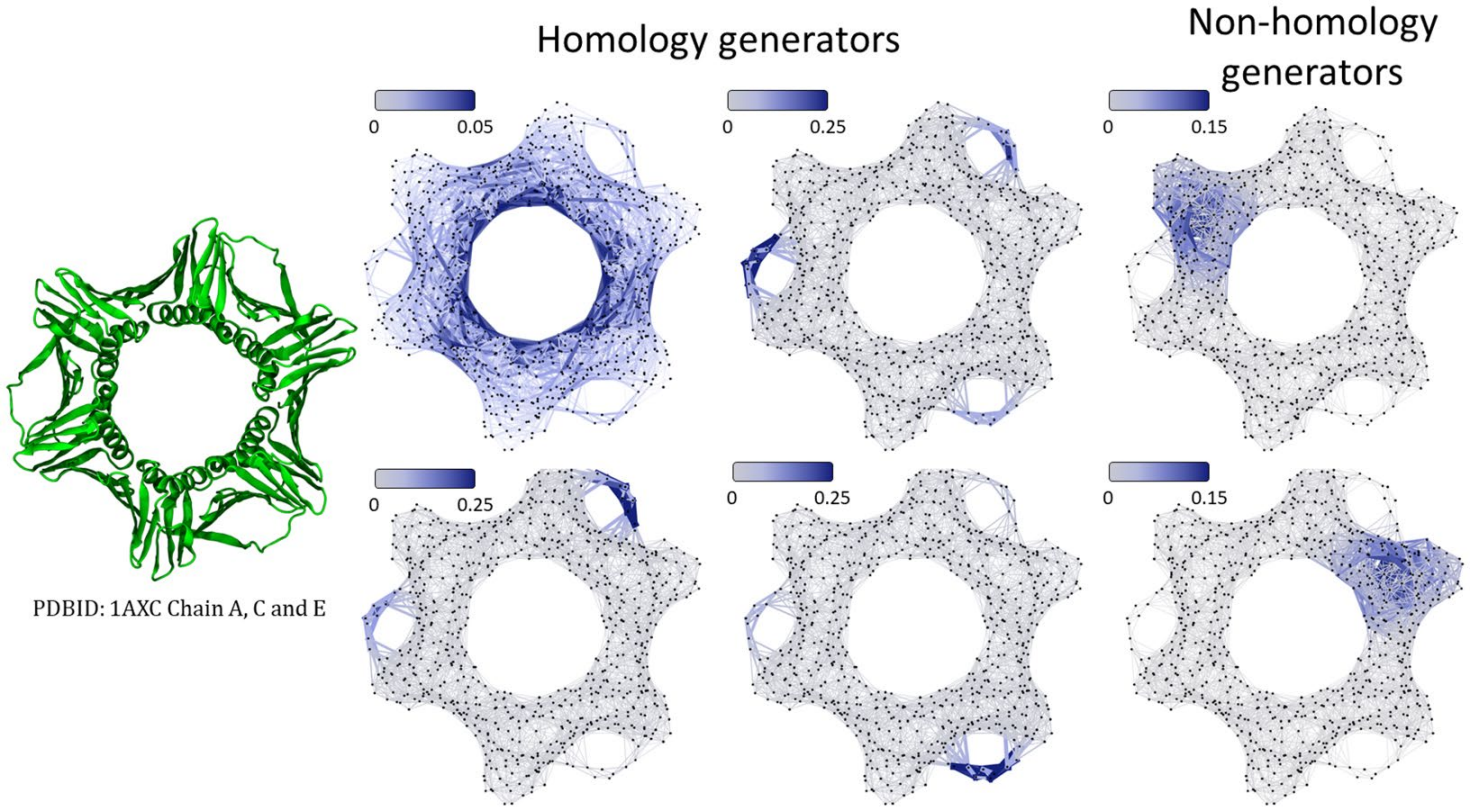
HL-based protein structure analysis for protein (ID:1AXC). Four 1D homology generators are from four zero eigenvalue related eigenvectors of 1D HL matrix. Two non homology generators are from two smallest nonzero eigenvalue related eigenvectors. Homology generators characterize loop and cycle structures, while non-homology generators indicate information about domains and communities.

The representation and characterization of biomolecular structures are of great importance for analyzing biomolecular functions. Among the various structural properties are biomolecular topological features, including rings, channels, cages, voids, etc. For instance, the closing and opening of ion channels are highly related to the channel structures. The virus capsids are cage-like structures with high symmetries. All these topological information can be well characterized by homology generators. Mathematically, eigenvectors of the zero eigenvalues of the *k*th HL matrices are *k*th homology generators.

Figure 4 illustrates the eigenvectors from 1-th HL matrix for protein (ID: 1AXC). There are four zero eigenvalues, i.e., the multiplicity of the zero eigenvalue is four. The corresponding eigenvectors characterize the cycle or loop structures. More specifically, if we plot the absolute value of the eigenvectors on the edges with large values represented by deep blue color, it can be seen that blue-colored edges are all concentrated around each cycle or loop. The nonzero eigenvalue related eigenvectors characterize clusters, domains, and communities. The two smallest nonzero eigenvalue related eigenvectors are depicted. Note that for each eigenvector, the large absolute values are all concentrated within a domain or a community.

#### Protein folding analysis

**Figure 5:**
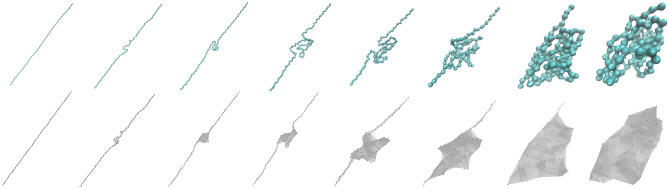
Eight configurations (after renumbering) extracted from the steered molecular dynamics simulation of Titin molecule, and their associated simplicial complexes. A cutoff distance of 11 Å is used to generate the Vietoris-Rips complex.

Here we use HodgeRank-based inconsistence to quantitatively measure the folding of protein configurations. We consider the Titin molecule. The trajectory data is obtained from the Steered molecular dynamics (SMD) simulation^65^. SMD simulations are designed to study the protein folding mechanism through an inverse unfolding process^65^. Essentially, a constant force or velocity is applied to one end of protein (with the other end fixed) to unfolded into a straight chain. In this way, various metastates can be observed from the dynamic process. We take 97 configurations equally from the simulation trajectory and renumber the sequence so that the last configuration (which is the straight chain) comes first and the first configuration (initial well-folded structure) comes last. Eight of these configurations are plotted in Fig. 5. Only $C_{\alpha }$ atoms are considered. It can be seen that, after renumbering, a protein folding process from a straight peptide chain to a well-folded 3D structure is observed. From these protein configurations, we can construct a series of simplicial complexes using the Vietoris-Rips complex. Figure 5 illustrates eight simplicial complexes generated from eight different Titin configurations. A cutoff distance of 11 Å is used to generate the Vietoris-Rips complex.

**Figure 6:**
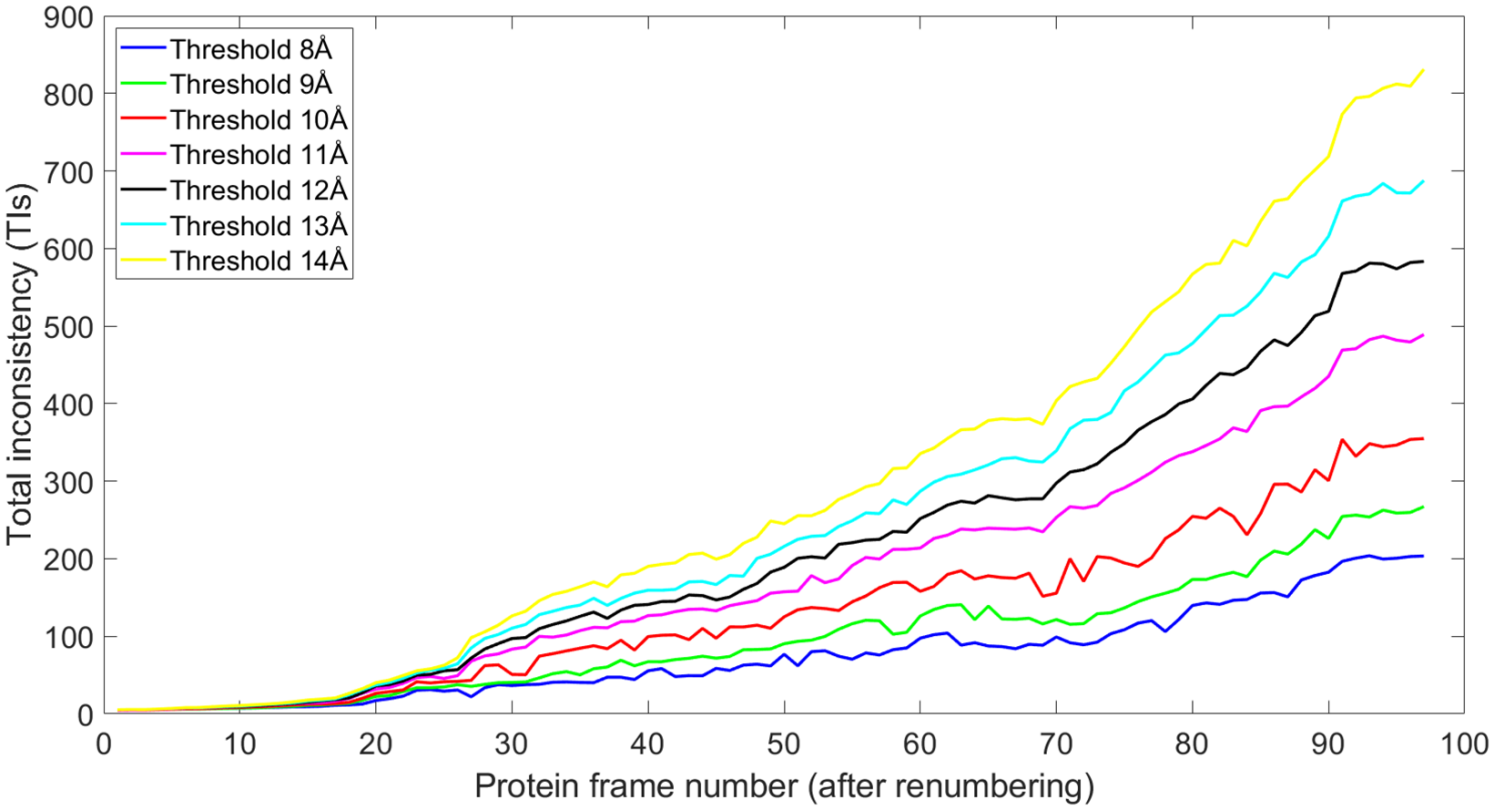
The illustration of total inconsistency (TI) for Titin configurations during the SMD simulations. A renumbering is considered so the first configuration is the last one in the SMD simulation. It can be seen that TIs are monotonically increasing when protein folds from a straight line to its 3D structures.

TI is used to measure the folding of protein structures. Since the Titin molecule has only a single chain, we take all the $C_{\alpha }$ atoms and rank them according to their amino acid sequence numbers. In this way, for two atoms $i_1$ and $i_2$ ($i_1<i_2$) with coordinate $\mathbf{r}_{i_1}$ and $\mathbf{r}_{i_2}$, if there exists an edge between them in a simplicial complex, their edge flow $Y_{[i_1, i_2]}=|\mathbf{r}_{i_1}-\mathbf{r}_{i_2}|$ according to Eq. (3). Furthermore, we can use the HodgeRank model and calculate TI for each configuration. Other than the cutoff distance of 11Å, we also consider other cutoff distances from 8Å to 14Å. Figure 6 illustrates the TIs of the 97 Titin configurations during the SMD simulation. As mentioned above, the renumbering is considered so the very first configuration corresponds to the straight line structure at the very end of SMD simulation. It can be seen that when Titin folds from a peptide chain to its 3D structure, the TIs increase monotonically with only small fluctuations. When Titin is a long unfolded peptide chain, TI is 0 as there is no curvedness or folding in the structure. The largest TI is obtained when Titin is well folded into its 3D structure. Moreover, with the enlargement of cutoff distance, the corresponding TI increases. This is due to the increasing size of associated simplicial complexes as cutoff distance increases. A larger cutoff distance ensures that the relations between atoms that are far away from each other are still well considered. More importantly, it can be seen clearly that as the increase of TI value, the fluctuations become smaller and smaller, and the TI curve becomes smoother. Even though a larger cutoff distance is preferred, the computational cost increases dramatically. Therefore, in our calculations, we do not consider a fully-connected simplicial complex, i.e., any $k+1$ atoms forming a *k*-simplex, instead, a median-sized cutoff distance is used. However, our TI still provides a suitable quantitative measurement for protein folding. Note that all these protein configurations have the same amount of atoms, therefore their corresponding AIs are of the same pattern as TIs. It is worth mentioning that the HodgeRank is based simplicial complex representation, if there is no 2-simplexes, all the curl flow terms will be zero.

#### DNA and chromatin folding analysis

**Figure 7:**
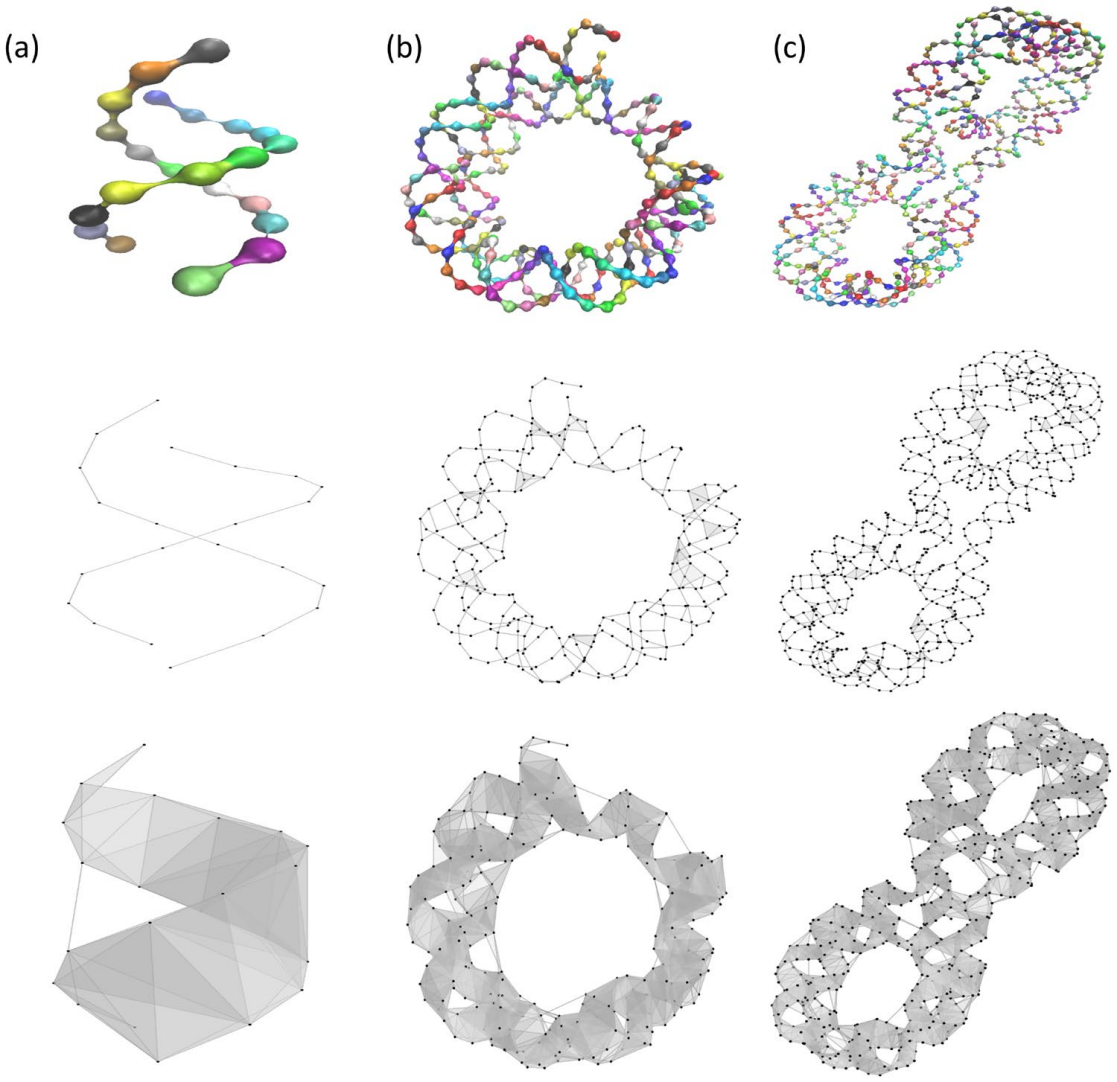
The illustration of three DNA structures, including DNA helix (**a**), nucleosome (**b**), and tetranucleosome (**c**), and their corresponding simplicial complexes at two different cutoff distances, i.e., 10 Å and 16 Å. The protein IDs for DNA helix, nucleosome, and tetranucleosome, are 330D, 6KVD and 1ZBB, respectively. The Vietoris-Rips complex is used.

We consider the folding of DNA at the atomic level. Three different DNA structures, including DNA helix, nucleosome and tetranucleosome (part of chromatin), are used in our analysis. Topologically, DNA helix is the preliminary structure, and can be folded into nucleosome and further into tetranucleosome. We consider only the phosphorus atoms in the three molecules and construct their simplicial complexes using Vietoris-Rips complex. The total numbers of phosphorus atoms for DNA helix, nucleosome and tetranucleosome, are 22, 291 and 692, respectively. Six different cutoff distances are used, including 10 Å, 12 Å, 14 Å, 16 Å, 18 Å and 20 Å. The DNA structures and the associated simplicial complexes are illustrated in Fig. 7. It can be seen that with a cutoff distance of 10 Å, no 2-simplexes are generated in the DNA helix structure. In contrast, with a cutoff distance of 16 Å, connected simplicial complexes are generated for all three structures.

The corresponding TIs and AIs, from the three DNA structures at different cutoff distances are illustrated in Table 1. Since the simplicial complex for the DNA helix structure at 10 Å has no 2-simplexes, there is no curl flow terms, i.e., all the $Y^c$ terms in Eqs. 1 and 2 are zero. Similarly, the $Y^h$ terms are also zero. In this way, the TI and AI for the DNA helix structure are all very close to 0. Similarly, at a cutoff of 12Å, both TI and AI are very close to 0 as no 2-simplexes are generated in DNA helix. Due to the folding of DNA chains, the TIs and AIs for both nucleosome and tetranucleosome structures are nonzero. Moreover, TIs for tetranucleosome are consistently larger that those of nucleosome. However, AIs for tetranucleosome are smaller that those of nucleosome at 10 Å. This is due to the reason that our AI is the average TI over the total number of atoms. From Fig. 7, it can be seen that the proportion of 2-simplexes over the total atom number for tetranucleosome is smaller than that of nucleosome, due to the missing 2-simplexes in the center linkage region. With the increase of cutoff distance, well-connected simplicial complexes are generated. The monotonic increase of TIs and AIs from DNA helix to nucleosome, and to tetranucleosome, can be observed clearly. There are highly consistent with the DNA folding patterns, indicating that both our TI and AI models are suitable for the description of curvedness, folding and compactness of biomolecular structures at molecular level.

**Table 1:** HodgeRank-based analysis of DNA folding at atomic level.

	TIs			AIs		
Cutoff	DNA helix	Nucleosome	Tetranucleosome	DNA helix	Nucleosome	Tetranucleosome
10 Å	0.0	279.3223	631.4930	0.0	0.9599	0.9126
12 Å	0.0	302.6626	734.4815	0.0	1.0401	1.0614
14 Å	28.9381	653.4245	1590.3249	1.3154	2.2454	2.2982
16 Å	33.9428	739.7261	1774.1848	1.5429	2.5420	2.5639
18 Å	46.7132	987.8988	2530.9683	2.1233	3.3948	3.6575
20 Å	63.1120	1308.3067	3191.6790	2.8687	4.4959	4.6123

There different DNA structures, including DNA helix (ID: 330D), nucleosome (ID: 6KVD) and tetranucleosome (ID:1ZBB), are considered. The simplicial complexes are generated using a series of different cutoff distances including, 10 Å, 12 Å, 14 Å, 16 Å, 18 Å and 20 Å.

#### Hodge decomposition-based Hi-C data analysis

**Figure 8:**
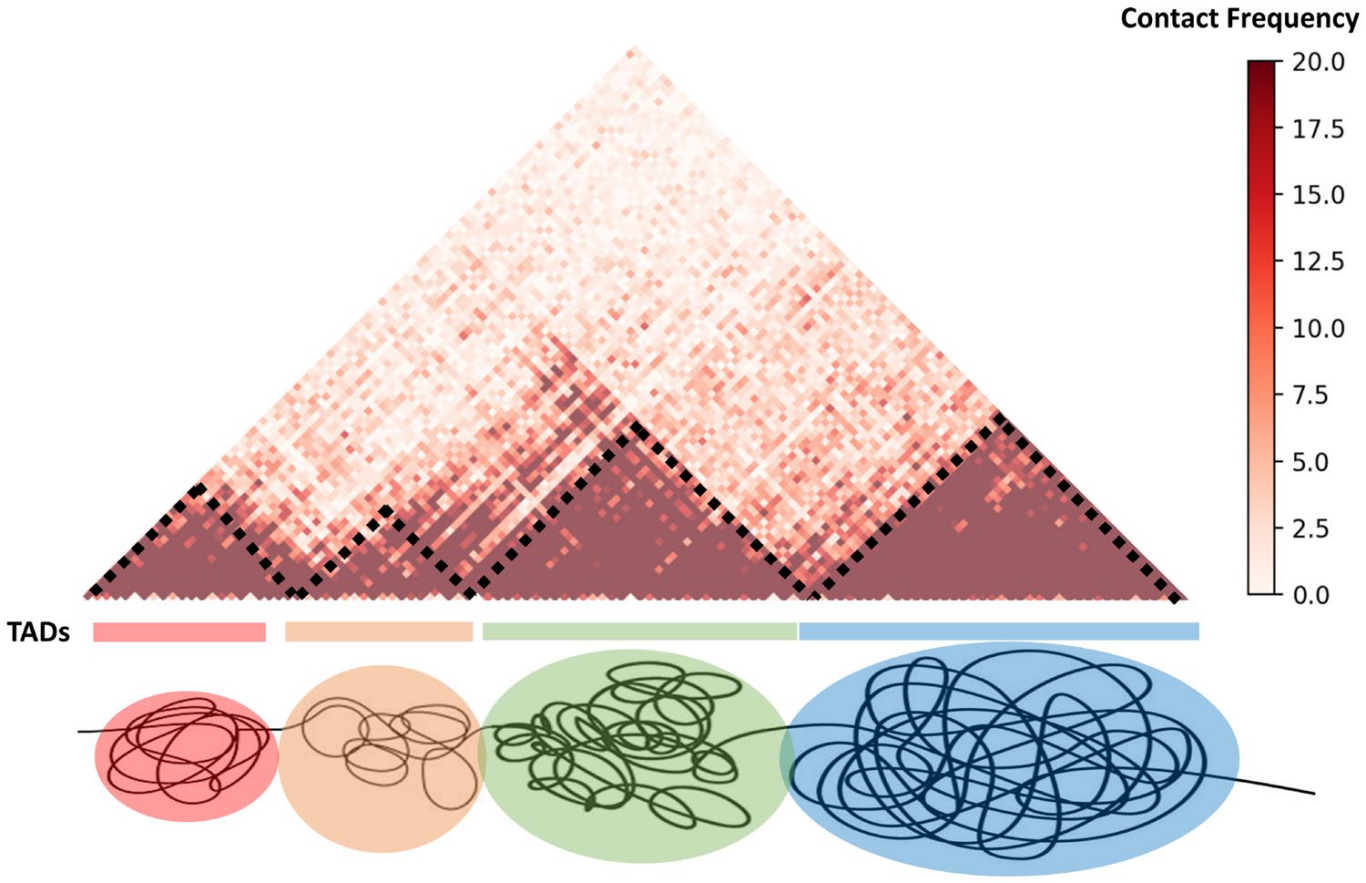
An illustration of topologically associating domains (TADs) for Hi-C data. The TAD is defined to be square region along the diagonal Hi-C maps with large contact values and a size of about 200 kilobases (Kb) to 2 megabases (Mb). The black dash lines mark the boundaries of TADs. Note that it is not always easy to clearly identify these “square regions”. For instance, it is also reasonable to believe that the two TADs in the middle region can be aggregated into one TAD.

Chromosomes have complicated hierarchial structures. Based on the analysis of Hi-C structures, it is believed that there are two possible types of structures (domains, subregions, etc), i.e., topologically associating domains (TADs) and genomic compartments. Computationally, TAD is defined to be the square region along the diagonal Hi-C maps with large contact values and a size of about 200 kilobases (Kb) to 2 megabases (Mb). Biologically, larger contact values mean these chromosomal loci (specific fixed positions on a chromosome) are close to each other, i.e., they are within a certain highly compacted/folded region. Figure 8 illustrates TAD regions in a Hi-C data. Geometrically, each TAD region (cartoon representation, not realistic experimental results) is believed to be a highly-packed region. The black dash lines mark the boundaries of TADs. However, the TAD is not mathematical rigorously defined, as it is not always easy to clearly identify the so-called “square regions”. For instance, its is also reasonable to believe that the two TADs in the middle region can be aggregated into one TAD. Due to the highly complicated hierarchial structure of chromosome, various algorithms have been developed to provide an approximation or estimation of TADs^52,60–63^, but a rigorous definition of TAD remains to be a problem.

**Figure 9:**
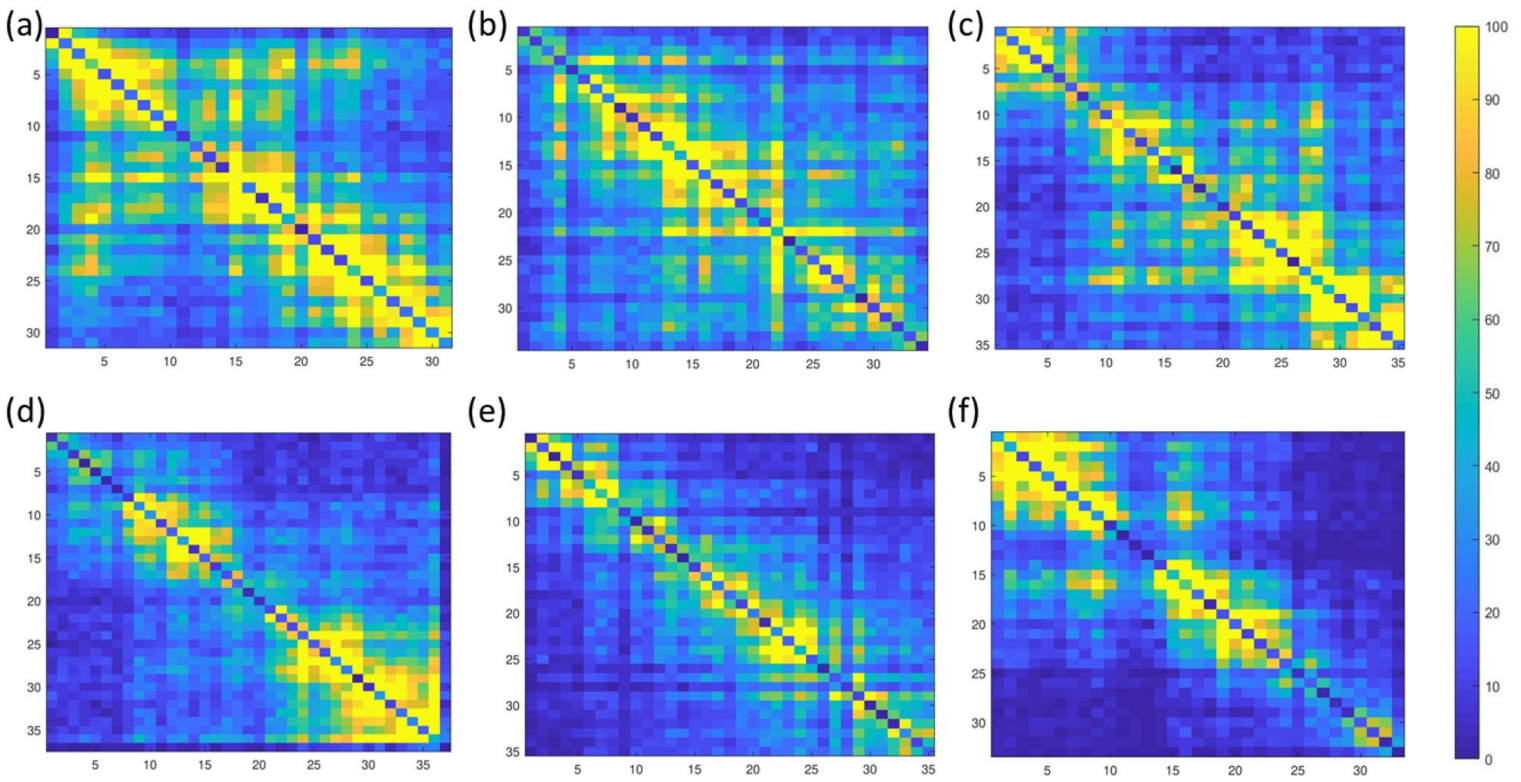
The illustration of six different TADs from Hi-C data of human ES (embryonic stem) chromosome 10. The contact frequency values are represented by colors. Bright yellow color indicate higher contact frequency, thus a short Eucledian distance between the two loci. As listed in Table 2, the AI values for TADs (**a**–**f**) are consistently decreasing, which is highly consistent with TAD patterns. We have also demonstrated six non-TADs in Fig. 10 and Table 3. It can be seen from the comparison that TADs tend to have more larger-contact values and their AI values are systematically larger than those from non-TADs.

In this subsection, we use HodgeRank-based AI index in the quantitative measurement topological associating domains calculated from Hi-C. Computationally, the entry $M_{i,j}$ of a Hi-C matrix *M* represents the contact frequencies between the *i*-th and *j*-th loci of the genome. The higher the contact frequencies between the *i*th and *j*th loci of the genome, the higher the probability that these two loci are closer to each other. Computationally, the distance *d*(*i*, *j*) between two loci *i* and *j* can be modeled as the $\alpha$-reciprocal of contact frequency,

$$\begin{aligned} d(i,j)=\frac{1}{M_{ij}^\alpha }. \end{aligned}$$

Here $\alpha$ is the power term and is usually chosen from the range of (0, 1). In our model, we consider the $\alpha$ value to be $\alpha =0.25$ and two cutoff distance $\gamma$ values, i.e., $\gamma =0.4$ and $\gamma =0.5$, to construct Hi-C matrix-based simplicial complexes. More specifically, if *d*(*i*, *j*) is smaller than the cutoff distance, an 1-simplex (edge) is formed between vertices *i* and *j*. Similarly, a 2-simplex (edge) is formed among three vertices *i*, *j*, and *k* if $d(i,j)\mathbin {{<}} \gamma$, $d(i,k)\mathbin {{<}}\gamma$, and $d(j,k)\mathbin {{<}}\gamma$. Since *d*(*i*, *j*) is not the direct experimental measurement for the distance between two loci, we consider two different types of the edge flows. The first type of edge flow is defined based on distance *d*(*i*, *j*) as follows,

4 $$\begin{aligned} Y_{[i, j]}= {\left\{ \begin{array}{ll} d(i,j) &{} i<j ~\mathrm{and}~ d(i,j)\mathbin {{<}}\gamma , \\ -d(i,j) &{} i>j ~\mathrm{and}~ d(i,j)\mathbin {{<}}\gamma . \end{array}\right. } \end{aligned}$$

A constant edge flow is used in our second model,

5 $$\begin{aligned} Y_{[i, j]}= {\left\{ \begin{array}{ll} 1 &{} i<j ~\mathrm{and}~ d(i,j)\mathbin {{<}}\gamma , \\ -1 &{} i>j ~\mathrm{and}~ d(i,j)\mathbin {{<}}\gamma . \\ \end{array}\right. } \end{aligned}$$

We call these two models as distance-based edge flow model and constant edge flow model respectively.

**Table 2:** HodgeRank-based TAD analysis.

AI	Distance-based edge flow		Constant edge flow	
	$\gamma =0.4$	$\gamma =0.5$	$\gamma =0.4$	$\gamma =0.5$
TAD(a)	3.8921	6.1816	4.0384	6.5773
TAD(b)	3.5610	6.5946	3.6861	6.9976
TAD(c)	3.0621	5.9346	3.1443	6.4456
TAD(d)	2.3628	5.3699	2.4740	5.7564
TAD(e)	1.6554	3.9307	1.7366	4.2958
TAD(f)	1.3350	3.4720	1.4520	3.8059

The AI values are used for the quantitative measurement of the folding within TAD. A larger AI value indicates more loops and high compactness of TAD region. In contrast, a lower AI value means less folding and less loops within TAD. Two different edge flow models, i.e., distance-based edge flow as in Eq. (4) and constant edge flow as in Eq. (4), are considered. Two different cutoff distances, i.e., $\gamma =0.4$ and $\gamma =0.5$, are used to construct Hi-C matrix-based simplicial complexes. Here TAD(a) to TAD(f) are TAD regions as illustrated in Fig. 9(a–f), respectively. For both models with two cutoff distance, the AI values decrease monotonically from TAD(a) to TAD(f), except a small inconsistency for at TAD( a) and TAD( b) at $\gamma =0.5$.

**Figure 10:**
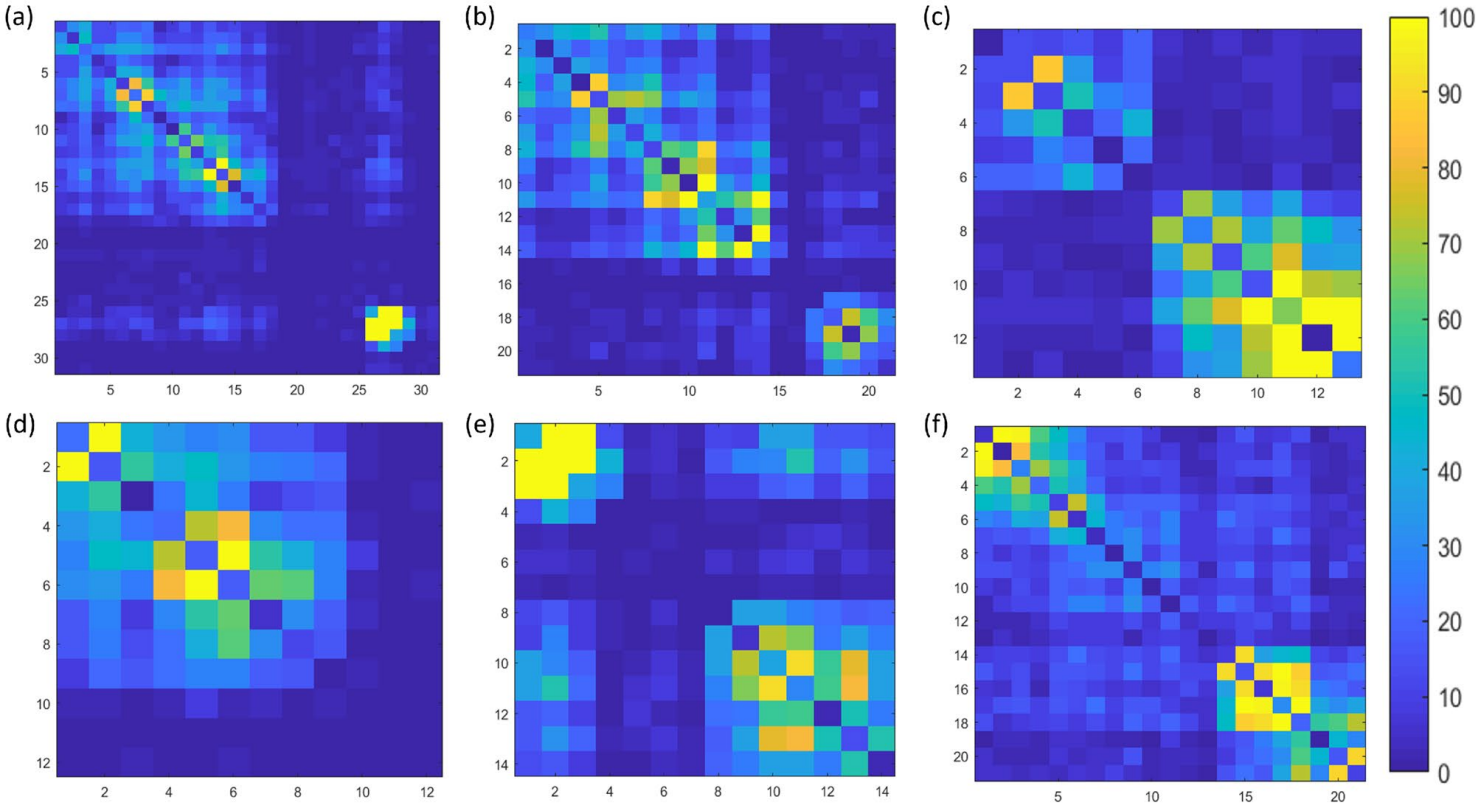
The illustration of six different non-TAD regions of human ES (embryonic stem) chromosome 10. The contact frequency values are represented by colors. Bright yellow color indicate higher contact frequency, thus a short Euclidean distance between the two loci. As listed in Table 3, the AI values for non-TAD regions (**a**–**f**) are dramatically low as compared to the AI values for TADs.

To test the performance of our two HodgeRank models for TAD analysis, we consider TAD regions obtained from human ES (embryonic stem) cells chromosome 10, using directionality index segmented by a Hidden Markov Model (HMM)^52^. The data has a resolution of 40,000 bp (base pairs) or 40 kb, i.e. each locus has a size of 40,000 bp. Six TAD regions are selected and depicted in Fig. 9. The values of contact frequency are represented by colors. A bright yellow color indicates a higher contact frequency, thus a short distance (between the two loci). We systematically evaluate the AIs for all six TAD regions using two edge flow models under two different cutoff distances as stated above. The results are listed in Table 2. To avoid confusion, TAD (a) to TAD ( f) are TAD regions as illustrated in Fig. 9, respectively. It can be seen that even though we use two different edge flow models, the pattern for AIs are highly consistent. That is the AI value monotonically decreases from TAD (a) to TAD (f), except for TAD( a) and TAD( b) at $\gamma =0.5$. In fact, TAD ( b) has a larger AI value than TAD ( a) in both edge flow models. Mathematically, there is no rigorous model to quantitatively measure the folding of TAD regions. However, if there are more larger contact frequency values, the loci are closer to each other (note that two adjacent loci has same distance), thus the TAD is more compact or folded. It can be observed that our AI values are highly consistent with the TAD patterns as seen in Fig. 9. Further, we consider six different non-TADs. These non-TAD regions are obtained from diagonal regions with lower contact values. Figure 10 illustrates these non-TAD regions and their AI values are listed in Table 3. It can be seen that lower AI values are systematically found for non-TADs than those for TADs.

**Table 3:** HodgeRank-based analysis on regions that are non-TADs.

AI	Distance-based edge flow		Constant edge flow	
	$\gamma =0.4$	$\gamma =0.5$	$\gamma =0.4$	$\gamma =0.5$
Non-TAD(a)	0.1354	1.3461	0.1594	1.4669
Non-TAD(b)	0.4656	1.3433	0.4866	1.4299
Non-TAD(c)	0.3069	0.7295	0.3320	0.8462
Non-TAD(d)	0.2722	1.0029	0.3090	1.1048
Non-TAD(e)	0.2450	0.8821	0.2500	0.9986
Non-TAD(f)	0.4802	1.2983	0.5187	1.4233

By non-TADs, we refer to the regions where contact frequencies are less or the regions where TADs are mostly not part of the region. The AI values are used for the quantitative measurement of the folding within non-TAD regions. Generally, low AI values were recorded due to the region having less contact frequency as compared to the AI values of TADs. Two different edge flow models, i.e., distance-based edge flow and constant edge flow, are considered. Two different cutoff distances, i.e., $\gamma =0.4$ and $\gamma =0.5$, are used to construct Hi-C matrix-based simplicial complexes. Here non-TAD(a) to non-TAD(f) are non-TAD regions as illustrated in Fig. 10a–f, respectively.

## Conclusion

Hodge theory characterizes the deep intrinsic relations of differential forms and provides a bridge between various areas in mathematics, including differential geometry, algebraic topology, and functional analysis. Here we considered both the Hodge Laplacian model and Hodge decomposition-based HodgeRank model for biomolecular data analysis. The HL-based spectral information, in particular, eigenvectors, are used for protein and DNA structure characterization. More specifically, homology generators are used for cycle and loop structure characterization. Non-homology related eigenvectors are used in clustering and community detection. Furthermore, we used the total and average inconsistency index from HodgeRank model to characterize the folding, compactness or curvedness of biomolecular structures and topological associated domains in Hi-C data. It has been found that our model can be used to quantitatively measure the folding within TADs. In the future, we will further explore the application of our HL-based clustering/classification, in particular, the homology-based and higher-order-simplex-based clustering/classfication. Moreover, we will study the relation of our AI values with genomic features such as histone modifications, coordinated gene expression, lamina, and DNA replication timing.

## Data Availability

All the data and codes in the paper are available at https://github.com/ExpectozJJ/Hodge-Theory.
